# Isolation and Epidemiology of Respiratory Bacteria From Unusual Sites: Demographic, Seasonal, and Geographic Distribution in a Laboratory-Based Study From a Tertiary Care Hospital in Karachi, Pakistan

**DOI:** 10.7759/cureus.80213

**Published:** 2025-03-07

**Authors:** Syeda R Zehra, Sobia M Khan, Samavia Mehmood, Joveria Farooqi, Kauser Jabeen

**Affiliations:** 1 Microbiology, Aga Khan University Hospital, Karachi, PAK

**Keywords:** demographic of unusual site isolation, epidemology, haemophilus parainfluenzae, invasive haemophilus influenzae disease, invasive streptococcus pneumoniae disease, laboratory-based study, respiratory pathogens, unusual anatomic sites

## Abstract

Introduction

Respiratory pathogens such as *Streptococcus pneumoniae*, *Haemophilus influenzae*, and *Haemophilus parainfluenzae* primarily infect respiratory and oropharyngeal sites. However, they are increasingly being isolated from unusual anatomic sites, such as urogenital and abdominal sources. These atypical occurrences necessitate an understanding of their epidemiology and clinical implications.

Methods

This study aimed to evaluate the seasonal prevalence, gender distribution, and clinical characteristics of *Streptococcus pneumoniae*, *Haemophilus influenzae*, and *Haemophilus parainfluenzae* isolated from nonrespiratory, unusual sites. Conducted at the Clinical Microbiology Laboratory of Aga Khan University Hospital in Karachi, Pakistan, the study included data from January 2021 to July 2023. Isolates from the urogenital tract, joints, skin and soft tissue, and other invasive samples from various body sites were included, while specimens from usual sites, such as blood, cerebrospinal fluid (CSF), and respiratory samples, were excluded. Analysis was performed after excluding isolates from unknown sources and duplicate samples from the same medical record number. A descriptive analysis was conducted on a small subset of inpatients with detailed clinical histories.

Results

Out of 2321 samples, 464 (20.0%) were excluded, and 1857 (80.0%) were analyzed, with 49 (2.6%) isolates from unusual sites. Isolation from unusual sites was significantly associated with female gender, the years 2021 and 2022, elderly age group, and Karachi origin (p < 0.05). *Haemophilus* species (68.6%) were more prevalent than *Streptococcus pneumoniae* (31.4%) in unusual sites. Seasonal distribution between usual and unusual sites was not statistically significant. A subset of 12 hospitalized patients with *Streptococcus pneumoniae* (n = 5), *Haemophilus influenzae* (n = 3), and *Haemophilus parainfluenzae* (n = 4) from unusual sites was analyzed. The average age was 44.9 years, with a 1:1 male-to-female ratio, and 75% had comorbidities. Infections involved bones/joints (n = 5), intraabdominal sites (n = 3), abscesses (n = 3), and the vagina (n = 1), with varied clinical presentations and empiric treatments. The average hospital stay was 6.25 days, and only one patient had bloodstream dissemination.

Conclusion

This study found a 2.6% isolation rate of *Streptococcus pneumoniae* and *Haemophilus* species from unusual sites, highlighting their clinical relevance. These findings will help microbiologists anticipate such infections and assist clinicians in optimizing empirical treatment strategies.

## Introduction

*Streptococcus pneumoniae* has a wide spectrum of disease manifestations, including nonbacteremic pneumonia, otitis media, and rhinosinusitis and invasive infections like bacteremic pneumonia, sepsis, meningitis, or invasive pneumococcal disease (IPD) [1]. Comorbidities such as asthma; chronic lung, kidney, and heart diseases; alcohol abuse; cigarette smoking; diabetes in immunocompetent hosts and HIV infection; anatomical or functional asplenia; solid or hematological malignancies; solid organ or hematopoietic cell transplantation; or sickle cell disease in immunocompromised hosts are associated with greater risk of progression to IPD [2]. Globally, pneumococcal infections account for 5% of all cause-child mortality under five years old, and the annual incidence of IPD ranges from 10 to 100 cases per 100,000 people in the United States and Europe. *Pneumococci* express over 90 capsular serotypes, exhibiting variable potential to cause disease, with some serotypes (one, two, four, five, seven F, eight, nine, 12F, 14, 16, 18C, 19A) being more invasive compared to others (three, sixA, sixB, 11A, 15B/C,19, and 23F) and serotypes 14 and 19A being the most common worldwide for IPD [3]. *Streptococcus pneumoniae* is the leading cause of community-acquired pneumonia and possesses several virulence factors that facilitate invasion beyond the respiratory tract. These factors contribute to its ability to cause infections in other sites, including the bloodstream and cerebrospinal fluid (CSF), leading to bacteremia and septicemia [4]. So isolation of these organisms from the respiratory system, blood, and CSF is considered its usual infection site, while isolation from all other sites of the body was considered unusual.

A number of studies have reported isolation of *Streptococcus pneumoniae* from unusual anatomic sites causing infections like urosepsis and pyelonephritis, tubo-ovarian abscess, abdominal wall abscesses, and mycotic aortic aneurysm [5-9]. Moreover, a retrospective study conducted on unusual manifestations of IPD demonstrated that the most common clinical presentation of uIPD was osteoarticular infection (36%), followed by gastrointestinal disease (18%). Additionally, infections with multidrug-resistant strains and all-cause mortality were higher in patients with unusual invasive pneumococcal disease (uIPD), which is usually caused by non-pneumococcal conjugate vaccine (non-PCV) 13 serotypes.

*Haemophilus influenzae* was the most common cause of meningitis before the introduction of vaccination. While vaccination has significantly reduced its incidence in meningitis cases, it remains a major pathogen responsible for upper and lower respiratory tract infections. Additionally, it can cause bacteremia and meningitis [10]. So isolation of these organisms from the respiratory system, blood, and CSF is considered their usual infection site, while isolation from all other sites of the body was considered unusual. Similarly, infections due to *Haemophilus species *have acquired growing interest during the last few decades, owing to the increasing infections with non-typeable *Haemophilus influenzae* strains and the accumulating sporadic reports of unusual infectious sites. A paper from Denmark reports that *Haemophilus species *were isolated from unusual anatomical sites in 80 patients, mostly adults, during a 15-year period (1973-1988). The majority of patients had soft tissue and bone infections followed by gastrointestinal infections, which included appendicitis, peritonitis following perforation of gastric ulcer, gall-duct infections, and an abscess in the stomach wall, while others had gynaecological foci: bartholinitis, salpingitis, and vaginitis [11]. 

The isolation of *Streptococcus pneumoniae* and *Haemophilus species* from uncommon sites underscore the importance of understanding their epidemiology and clinical implications. A comprehensive analysis of these infections will enable microbiologists to better anticipate the presence of these pathogens in unusual anatomical sites. Additionally, this knowledge will aid clinicians in formulating effective empirical treatment strategies and optimizing clinical management. 

The primary objective was to determine the frequency of isolation of typical respiratory pathogens from unusual sites out of all the patients who are culture-positive for them with respect to season, geographic location, age, and gender. The secondary objective is to assess presenting complaints, comorbidities, length of hospitalization, history of previous respiratory illness, empiric treatment given, and the presence of the same organism in blood culture in a subset of hospitalized patients with infections caused by these pathogens.

## Materials and methods

This was a cross-sectional study conducted at the Clinical Microbiology Laboratory, Aga Khan University Hospital in Karachi, Pakistan. Clinical reports of specimens showing growth of *Streptococcus ​​​​​​pneumoniae*, *Haemophilus influenzae*, and *Haemophilus​​​parainfluenzae *from January 2021 to July 2023 were extracted from the laboratory database and included in the study. For the isolation of *Haemophilus influenzae, Haemophilus parainfluenzae*, and *Streptococcus pneumoniae*, we used chocolate agar and sheep blood agar. Identification was performed using conventional biochemical methods, and antimicrobial susceptibility testing was conducted using the disk diffusion method according to the CLSI guidelines. Isolates were categorized in “usual” and “unusual” sites: Usual sites were defined as anatomical sites from which *Streptococcus*​​​​​* pneumoniae*, *Haemophilus influenzae*, and *Haemophilus​​​​​​​ parainfluenzae* are commonly isolated, including blood, CSF, and respiratory specimens. Unusual sites were defined as anatomical sites excluding usual sites from the urogenital system, joints, skin, and soft tissue and other invasive samples. Age groups were categorized as children, 0-18 years; adults, 19-54 years; and elderly, 55+ years. Analysis was performed after excluding isolates from unknown sources and more than one sample from the same medical record number. A descriptive analysis was done on a small subset of inpatients whose history was present in detail.

The primary outcome was to determine the frequencies with respect to season of occurrence, age, gender, and city of patients with infections caused by *Streptococcus ​​​​​​pneumonia, Haemophilus​​​​​​​ influenzae*, and* Haemophilus​​​​​​​ parainfluenzae* in unusual anatomic sites. The secondary outcome was the description of clinical and treatment-related factors, comorbidities, previous respiratory illnesses, and patient outcomes in a subset of patients.

Statistical analysis was conducted using IBM SPSS Statistics for Windows, Version 23 (Released 2015; IBM Corp., Armonk, New York, United States). The distribution of organisms in usual and unusual sites was assessed through frequency calculations. Associations with geographical location, winter season, age groups, and gender were evaluated using Fisher’s exact test and the Chi-square test. Logistic regression was performed to identify factors associated with the isolation of these pathogens in unusual sites, considering variables with p-values of <0.02 in the primary analysis. Additionally, a sub-analysis was conducted for each organism isolated from unusual sites to examine its association with geographical location, season, age groups, and gender.

## Results

**Figure 1:**
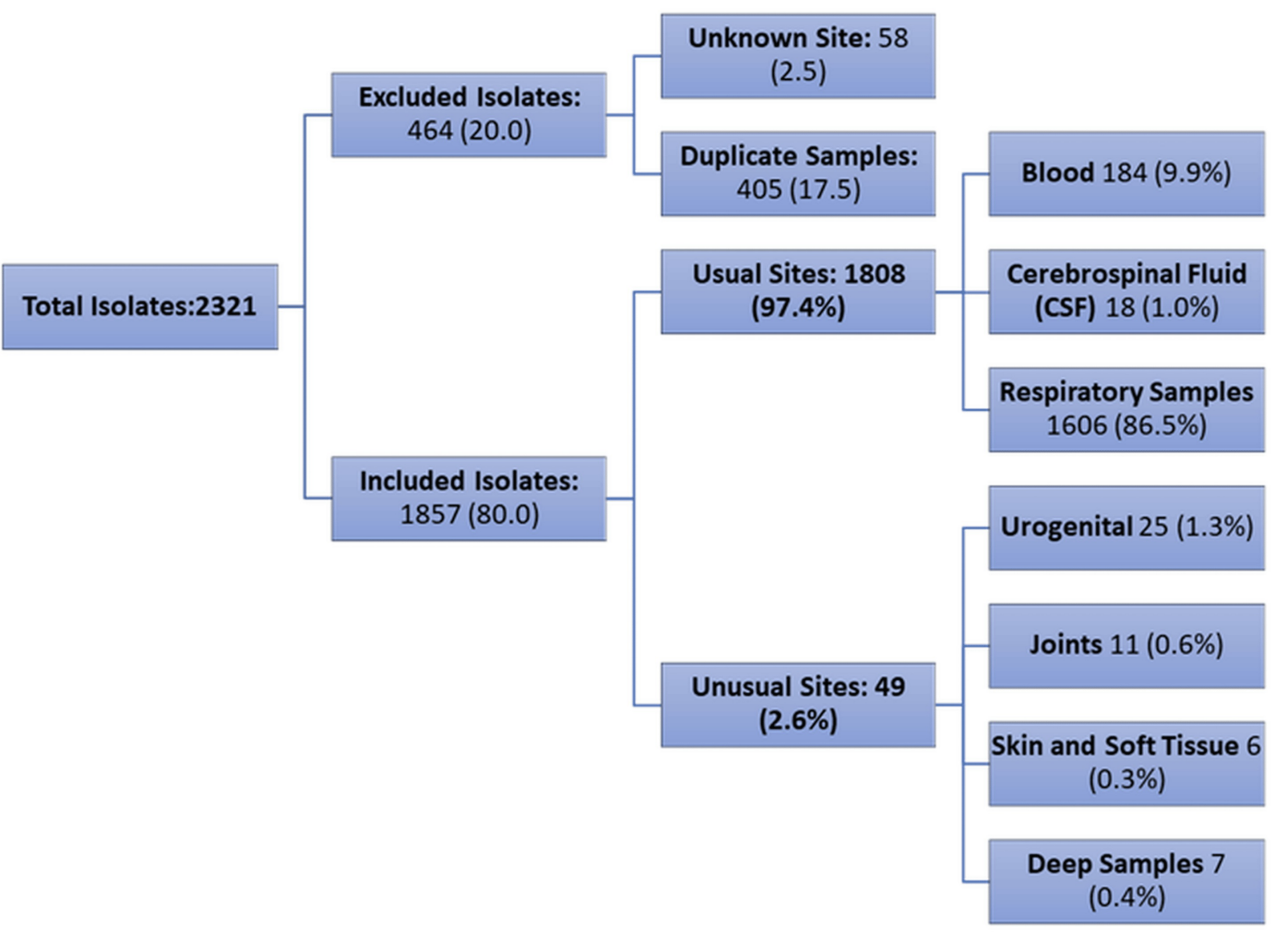
This figure illustrates the distribution of 1857 isolates included in the final analysis A majority (97.4%, n = 1808) were from usual sites, with respiratory samples being the most frequent (86.5%, n = 1606), followed by blood (9.9%, n = 84) and cerebrospinal fluid (1.0%, n = 18). Unusual sites accounted for 2.6% (n = 49) of isolates, predominantly from urogenital samples (1.3%, n=25), followed by joint (0.6%, n = 11), skin and soft tissue (0.3%, n = 6), and deep tissue samples (0.4%, n = 7)

Out of 2321 samples, 464 (20.0%) were excluded: 58 (2.5%) from unknown sites and 405 (17.5%) duplicate samples. A total of 1857 (80.0%) isolates were included in the final analysis. Among these, 1808 (97.4%) were from usual sites, predominantly respiratory samples (1606, 86.5%), followed by blood (84, 9.9%) and CSF (18, 1.0%). Unusual sites accounted for 49 (2.6%) isolates, with the majority from urogenital samples (25, 1.3%), followed by joint (11, 0.6%), skin and soft tissue (6, 0.3%), and deep tissue samples (7, 0.4%) (Figure 1).

The isolation of* Streptococcus* ​​​​​*pneumoniae, Haemophilus* *influenzae, *and *Haemophilus parainfluenzae* from usual and unusual sites demonstrated statistically significant associations with gender, year of isolation, different age groups, and geographic location (p-value < 0.05) (Table 1). Females, the years 2021 and 2022, adult (18-54 years) and elderly age group, and sample origin from within Karachi were all positively associated with isolation from unusual sites.

**Table 1:** Key factors in usual and unusual site infections † A further analysis was done to find the association with the sample site and gender, and a significant association was found between female gender and isolates from the urogenital site (OR: 9.77, 95% CI: 1.43–66.86, p = 0.02) This table summarizes demographic and epidemiological factors associated with unusual infection sites, including gender, organism prevalence, season, year, age, and geography, using the Chi-square test. Gender (p = 0.00) and age (p = 0.04) play a significant role in infection in unusual sites. Unusual site infections have declined over time (p = 0.01), with unusual site infections being more common in samples from Karachi compared to other regions (p < 0.01). Organism type and seasonal variation do not show a statistically significant difference

Category	Usual (1808)	Unusual (49)	Total (1857)	p-value
Gender				0.00*
Male	1129 (62.4%)	16 (32.7%)	1145 (61.7%)	
Female †	679 (37.6%)	33 (67.3%)	712 (38.3%)	
Organism				0.22
Streptococcus pneumoniae	533 (29.5%)	16 (30.6%)	549 (29.6%)	
Haemophilus influenzae	821 (45.4%)	17 (34.7%)	838 (45.1%)	
Haemophilus parainfluenzae	453 (25.1%)	17 (34.7%)	470 (25.3%)	
Season				1.00
Winter	1170 (64.7%)	32 (65.3%)	1202 (64.7%)	
Other seasons	638 (35.3%)	17 (34.7%)	655 (35.3%)	
Year				0.01*
2021	440 (24.3%)	19 (38.8%)	459 (24.7%)	
2022	737 (40.8%)	22 (44.9%)	759 (40.9%)	
2023	631 (34.9%)	8 (16.3%)	639 (34.4%)	
Age group				0.04*
Children (0-18 yrs)	366 (20.2%)	9 (18.4%)	375 (20.2%)	
Adults (19-54 yrs)	722 (39.9%)	28 (57.1%)	750 (40.4%)	
Elderly (55+ yrs)	720 (39.8%)	12 (24.5%)	732 (39.4%)	
Province				0.00*
Pakistan outside Karachi	711 (39.3%)	9 (18.4%)	720 (38.8%)	
Karachi	1097 (60.7%)	40 (81.6%)	1137 (61.2%)	

The distribution of organisms and seasonal variation did not demonstrate statistical significance. *Streptococcus ​​​​​pneumoniae*,* Haemophilus ​​​​​​influenzae,* and *Haemophilus parainfluenzae* were isolated from 533 (29.5%), 821 (45.4%), and 453 (25.1%) of usual site cases, respectively, compared to 16 (30.6%), 17 (34.7%), and 17 (34.7%) of unusual site cases. Seasonal distribution showed that 1170 (64.7%) of usual site cases occurred in winter and 638 (35.3%) in other seasons, whereas 32 (65.3%) and 17 (34.7%) of unusual site cases were reported during winter and other seasons, respectively (Table 1).

Multivariable analysis revealed that females had significantly higher odds of having infections from unusual sites than males (OR: 3.36, 95% CI: 1.824-6.209, p = 0.00). Year-wise comparison showed that infections in 2021 and 2022 had significantly higher odds of being from unusual sites compared to those reported in 2023 (OR: 3.456, 95% CI: 1.486-8.033, p = 0.004) and (OR: 2.226, 95% CI: 0.977-5.071, p = 0.057), respectively. Geographical analysis indicated that cases from Karachi had significantly higher odds of being from unusual sites compared to other regions (OR: 3.598, 95% CI: 1.707-7.581, p = 0.001). Age group comparisons demonstrated that the elderly had higher odds of infection at unusual sites than the children (OR: 2.537, 95% CI: 1.258-5.116, p = 0.009), while the association with adults compared to children did not reach statistical significance (OR: 1.376, 95% CI: 0.569-3.327, p = 0.479) (Table 2).

**Table 2:** Multivariate analysis This table presents the results of multivariate analysis assessing the association between various factors with isolation of *Streptococcus pneumoniae*, *Haemophilus influenzae,* and *Haemophilus parainfluenzae* from unusual sites. The odds ratio (OR), the 95% confidence interval (CI), and the corresponding p-values are provided for each comparison. A statistically significant association was observed for gender, year, age, and geographical site with females, years 2021 and 2022, isolates from Karachi, and the elderly patients are at higher odds of being from unusual sites

Variable	Odds ratio	95% CI (lower-upper)	p-value
Female vs male	3.36	1.824-6.209	0.00
2023 vs 2021	3.456	1.486-8.033	0.004
2023 vs 2022	2.226	0.977-5.071	0.057
Karachi vs Pakistan, other than Karachi	3.598	1.707-7.581	0.001
Adults vs children	1.376	0.569-3.327	0.479
Elderly vs children	2.537	1.258-5.116	0.009

A further overall analysis of 51 unusual cases revealed that *Haemophilus* species were significantly more prevalent (68.63%) than *Streptococcus* *pneumoniae *(31.37%). *Streptococcus *​​​​​​*pneumoniae* was isolated in six (40.0%) and nine (60.0%) males and females, respectively. It was detected in four (26.7%), six (40.0%), and five (33.3%) cases in the years 2021, 2022, and 2023, respectively. Geographically, 10 (66.7%) cases were reported from Karachi and five (33.3%) from Pakistan outside Karachi. Seasonal distribution showed seven (46.7%) cases in winter and eight (53.3%) in other seasons. By site of isolation, *Streptococcus *​​​​​​*pneumoniae* was identified in five (33.3%) from urogenital samples, four (26.7%) from joints, four (26.7%) from skin and soft tissue, and two (13.3%) from deep samples.

*Haemophilus *species were isolated in 10 (29.1%) and 24 (70.6%) males and females, respectively. The organism was identified in 15 (44.1%), 16 (47.1%), and three (8.8%) cases in the years 2021, 2022, and 2023, respectively. A total of 30 (88.2%) cases were reported from Karachi and four (11.8%) from Pakistan outside Karachi. Seasonal distribution showed 10 (29.4%) cases in winter and 24 (70.6%) in other seasons. By site of isolation, *Haemophilus *species were identified in 20 (58.8%) from urogenital samples, seven (20.6%) from joints, two (5.9%) from skin and soft tissue, and five (14.7%) from deep samples. None of the above comparisons showed statistical significance (Table 3).

**Table 3:** Comparison of Streptococcus pneumoniae and Haemophilus species in unusual sites The table presents the distribution of *Streptococcus pneumoniae* and *Haemophilus* species isolated from unusual sites, stratified by gender, season, year, age group, geographic location, and site of infection. Data are expressed as counts and percentages. Fisher's exact test was applied. No statistically significant differences were observed across these variables (p > 0.05)

Category	*Streptococcus pneumoniae *(16)	*Haemophilus* species (33)	Total	p-value
Gender				0.52
Male	6 (40.0%)	10 (29.1%)	16 (32.7%)	
Female	9 (60.0%)	24 (70.6%)	33 (67.3%)	
Season				0.33
Winter	7 (46.7%)	10 (29.4%)	17 (34.7%)	
Other seasons	8 (53.3%)	24 (70.6%)	32 (65.3%)	
Year				0.09
2021	4 (26.7%)	15 (44.1%)	19 (38.8%)	
2022	6 (40.0%)	16 (47.1%)	22 (44.9%)	
2023	5 (33.3%)	3 (8.8%)	8 (16.3%)	
Age group				0.27
Children (0–18 yrs)	4 (26.7%)	5 (14.7%)	9 (18.4%)	
Adults (19–54 yrs)	6 (40.0%)	22 (64.7%)	28 (57.1%)	
Elderly (55+ yrs)	5 (33.3%)	7 (20.6%)	12 (24.5%)	
Province				0.11
Pakistan (outside Karachi)	5 (33.3%)	4 (11.8%)	9 (18.4%)	
Karachi	10 (66.7%)	30 (88.2%)	40 (81.6%)	
Site				0.15
Urogenital	5 (33.3%)	20 (58.8%)	25 (51.0%)	
Joints	4 (26.7%)	7 (20.6%)	11 (22.4%)	
Skin & soft tissue	4 (26.7%)	2 (5.9%)	6 (12.2%)	
Deep samples	2 (13.3%)	5 (14.7%)	7 (14.3%)	

The secondary objective of this study was to assess the clinical presentation, comorbidities, empiric treatment, and history of previous respiratory illnesses in a subset of hospitalized patients infected with *Streptococcus pneumoniae, Haemophilus* *influenzae*, or *Haemophilus parainfluenzae*. Descriptive analysis was performed on 12 inpatients meeting the inclusion criteria.

Of these 12 patients, five were infected with *Streptococcus* *pneumoniae*, three with *Haemophilus* *influenzae*, and four with *Haemophilus parainfluenzae.* The average age of the patients included in this analysis was 44.9 years. The male-to-female ratio was 1:1 (50% each). Comorbid conditions were present in nine patients: three patients had diabetes mellitus, five had hypertension, two had hypothyroidism, one had neurofibroma, one had carcinoma rectum, and one had nephrotic syndrome. Three patients had no documented comorbidities.

The infections were associated with various unusual anatomical sites, including five cases involving bones and joints, three cases of intraabdominal infections, three abscesses (psoas, liver, fascial), and one vaginal infection. Additionally, three patients had a documented history of pleural effusion. The clinical manifestations varied, with presentations including joint pain and swelling (shoulder, knee, foot), abdominal pain and distension, left hand cellulitis, vaginal leaking, fever with shortness of breath, and discharge from infected wounds.

Empiric treatment varied among patients, with antibiotics such as vancomycin, clindamycin, linezolid, ciprofloxacin, levofloxacin, amoxicillin-clavulanic acid, cefixime, ceftriaxone, piperacillin-tazobactam, and meropenem being administered. One patient did not receive empiric antibiotic therapy, while one patient’s status was unknown. The average length of hospital stay was 6.25 days (range: 3-15 days). Notably, only one patient had *Streptococcus ​​​​​​ pneumoniae* isolated from blood cultures, indicating systemic dissemination.

Descriptive data of 12 inpatients with *Streptococcus* ​​​​​​*pneumoniae* and *Haemophilus *species isolated from unusual sites highlighted diverse clinical presentations, comorbidity profiles, and infection sites of *Streptococcus* *pneumoniae*, *Haemophilus* ​​​​​​*influenzae*, and *Haemophilus* *parainfluenzae* in hospitalized patients (Table 4).

**Table 4:** Descriptive data PPROM: preterm premature rupture of the membrane; *Patient who had positive blood culture with the same organism. †Patients who had documented respiratory illness (pleural effusion) Descriptive data of 12 inpatients with *Streptococcus pneumoniae* and *Haemophilus species* isolated from unusual sites

Diagnosis	Age	Gender	Organism isolated	Clinical manifestations	Comorbidities	Length of hospitalization (days)	Empiric treatment
Left hand cellulitis	11 months	F	S. pneumoniae	Left hand swelling	None	4	Vancomycin, clindamycin
Neurofibroma	12	M	H. parainfluenzae	Fascial abscess	Neurofibroma	6	Not known
Nephrotic syndrome	13	M	S. pneumoniae	Psoas abscess	Steroid-resistant nephrotic syndrome	3	Amoxicillin + clavulanic acid
PPROM	21	F	H. influenzae	Vaginal leaking at 38 weeks and 2 days gestation	None	5	Ciprofloxacin
Left septic arthritis*	48	F	S. pneumoniae	Left knee pain	Diabetes mellitus	7	Ceftriaxone
Infected abdominal mesh	50	M	H. parainfluenzae	Discharge from abdominal wound	Diabetes mellitus, hypertension	4	Linezolid
Left diabetic foot	51	F	H. influenzae	Left foot swelling	Diabetes mellitus	4	Vancomycin, ceftriaxone
Right shoulder pain	56	F	S. pneumoniae	Right shoulder pain	Hypertension	3	Meropenem, levofloxacin
Biliary sepsis †	67	M	H. influenzae	Abdominal pain and distension	Diabetes mellitus, hypothyroidism, hypertension	5	Clindamycin, ciprofloxacin
Dengue shock syndrome, liver abscess †	70	F	H. parainfluenzae	Fever, shortness of breath	None	9	None
Left shoulder septic arthritis	75	M	S. pneumoniae	Left shoulder pain and swelling	Hypertension, hypothyroidism	6	Piperacillin, tazobactam
Pneumoperitoneum and peritonitis †	75	M	H. parainfluenzae	Abdominal pain	Hypertension, carcinoma rectum	15	Cefixime

## Discussion

*Streptococcus* *pneumoniae*, a common colonizer of the nasopharynx, has developed intricate mechanisms to invade deeper tissues, leading to severe infections beyond pneumonia and bacteraemia. Specific proteases play pivotal roles in enhancing adhesion, colonization, and tissue invasion [12]. Similarly, *Haemophilus* ​​​​​​*influenzae *transitions from a commensal in the human respiratory tract to an invasive pathogen under specific conditions. Encapsulated strains utilize their polysaccharide capsules to evade immune defences, resisting phagocytosis and complement-mediated lysis. Infections caused by encapsulated strains are particularly prevalent among older adults [13-15]. Given that most cases were observed in elderly patients, further studies are warranted to investigate the molecular types and virulence factors contributing to these infections.

Studies have consistently shown a higher incidence of pneumococcal disease in males across all age groups. This increased susceptibility in males may be attributed to immunological differences, including X chromosome inactivation, sex hormone influence, and higher prevalence of chronic conditions such as cardiovascular disease, malignancies, and smoking [16]. Behavioral factors, such as smoking, further contribute to the elevated risk in men [14]. While our study found more isolates from usual sites (blood, CSF, and respiratory specimens) in males, females had a higher prevalence of *Streptococcus* *pneumoniae* and *Haemophilus species* isolates from unusual sites. This can be attributed to the fact that the majority of unusual site samples were from the urogenital system, particularly high vaginal swabs (22, 88%), compared to only 3 (12%) urethral samples. This association was statistically significant (OR: 9.77, 95% CI: 1.43-66.86, p = 0.02).

This study demonstrated that 60.7% of isolates originated from Karachi, with 81.6% of unusual site isolates also from this region. The predominance of isolates from Karachi can be explained by the location of the laboratory in Karachi, allowing the fastidious organisms to survive over a shorter transit time [15,17]. Samples from sites outside Karachi usually take more than 24 hours to reach and be processed in the lab, and this time lapse can explain the low yield of these organisms from samples from outside Karachi. Other possible contributing factors include the high population density of Karachi, which may facilitate the transmission of infections, and comparatively better access to healthcare compared to rural areas or the northern provinces of Pakistan. Furthermore, our laboratory has a larger number of collection points in Karachi than in other regions, leading to a relatively higher sample collection from this city.

Globally, the epidemiological patterns of various pathogens experienced significant shifts during the COVID-19 pandemic. A study from England reported that from July to December 2021, the incidence of IPD in children under 15 years of age was higher (1.96 per 100,000 children) compared to the same period in 2020 (0.7 per 100,000 children) and the prepandemic years 2017-2019 (1.43 per 100,000 children). This increase may be attributed to a larger proportion of susceptible children who had limited exposure to pneumococci during the lockdown [18]. In the fall of 2021, Quebec, Canada, reported a resurgence of IPD in children under five years old, which coincided with an unusual surge in RSV infections. In contrast, no rebound was observed in older age groups, where IPD cases appeared to align more closely with influenza activity [19]. Our study aligns with these observations, as the highest number of unusual site isolates were recorded in 2021.

As true for usual sites, unusual site isolates were also more prevalent in the elderly population, as demonstrated by our study. This can be explained by several factors, such as the greater prevalence of chronic conditions, such as cardiovascular and renal diseases, malignancies, and immunosuppression in this population [15,17,20].

Despite these findings, the study has several limitations. It is a single-center study from a laboratory network centered in Karachi, so the geographical distribution of the data is skewed. This study is a single-center analysis, which limits its generalizability to broader populations and healthcare settings. Future prospective multicenter studies are needed to better account for potential confounders such as comorbidities and antibiotic use, allowing for more robust conclusions and improved generalizability of findings. No significant association was found between organism type and site type, potentially due to the small sample size for unusual sites. These limitations underscore the need for larger, more regionally representative datasets to improve the generalizability of findings. Future research should explore the mechanisms underlying observed gender and age differences, as well as unique factors contributing to infections at unusual sites. Molecular-based diagnostics, which do not rely on organism viability, can provide an accurate representation of the geographical distribution. Serotyping *Streptococcus* *pneumoniae *and *Haemophilus* ​​​​​​*influenzae* and analyzing their virulence factors may help explain the decline in isolation observed in 2023. Surveillance and diagnostic capabilities, along with targeted epidemiological studies, are essential to address these gaps and improve patient outcomes. By building on the insights gained from this study, researchers can advance the understanding of the epidemiology and pathogenesis of these organisms, fostering more effective prevention and treatment strategies.

## Conclusions

This study found a 2.6% isolation rate of respiratory pathogens, *Streptococcus* *pneumoniae* and *Haemophilus* species, from unusual body sites. The findings of this study will guide microbiologists to better anticipate the presence of these pathogens in unusual anatomical sites. Additionally, this knowledge will aid clinicians in formulating effective empirical treatment strategies, modifying empiric treatment based on local antimicrobial resistance data, and optimizing clinical management.
